# Outcomes of Geriatric Hip Fractures in a Tertiary Referral Center in Malaysia During the COVID-19 Pandemic

**DOI:** 10.7759/cureus.40479

**Published:** 2023-06-15

**Authors:** Mohammed Harris Anwarali Khan, Ren Yi Kow, Sasidaran Ramalingam, Azlan Sofian, Jade Pei Yuik Ho, Kamaljeet Singh Jaharan Singh, Jeffrey Jaya Raj, Kunalan Ganthel@Annamalai, Fazir Mohamad

**Affiliations:** 1 Department of Orthopaedics, Hospital Kuala Lumpur, Kuala Lumpur, MYS; 2 Department of Orthopaedics, Seberang Jaya Hospital, Seberang Jaya, MYS; 3 Department of Orthopaedics, Traumatology and Rehabilitation, International Islamic University Malaysia, Kuantan, MYS; 4 Department of Orthopaedic Surgery, Tuanku Ja'afar Hospital, Seremban, MYS; 5 Department of Orthopaedic Surgery, Kuala Lumpur General Hospital, Kuala Lumpur, MYS

**Keywords:** trivial fall, hip joint, bipolar hemiarthroplasty, total hip replacement (thr), neck of femur fractures, geriatric hip fracture

## Abstract

Introduction

With the advancing age of the population, there are an increasing number of patients with geriatric hip fractures. Despite the advancement of surgical knowledge and improvement of implant designs to treat geriatric hip fractures, mortality and morbidity remain high among these frail patients. In conjunction with the COVID-19 pandemic, the collateral damage dealt to these patients remains unknown as scarce resources are funneled to deal with the pandemic. This study is geared to investigate the surgical outcomes of patients with geriatric hip fractures who were admitted during the initial phase of the COVID-19 pandemic.

Methods

This retrospective study was carried out at Hospital Kuala Lumpur, the largest public hospital in the capital of Malaysia, from March 1, 2020, to March 1, 2021. All patients of age 60 years and above were screened for suitability. Only patients who had undergone surgical intervention during the study period were included in this study. Patients’ demographic data, mechanism of injury, waiting time for surgery, type of surgery, complications and ambulatory status were obtained from the medical records. Univariate analysis was performed to determine the factors associated with complications as well as the post-operative ambulatory status of the patients.

Results

A total of 52 patients were included in this study, with a median age of 72 years. The majority of the patients were Chinese (n=21, 40.4%). This was followed by Malay and Indian (n=14, 26.9% each) and other ethnicity (n=3, 5.8%). More than three-quarters of the patients had a trivial injury such as a fall due to a miss-step (n=16, 30.8%) and slip (n=16, 30.8%) and a fall due to dizziness (n=8, 15.4%). Only 12 patients (23.1%) sustained hip fractures due to trauma. The median time to surgery for these patients was 5 days (interquartile range: 4 days). Most of these patients underwent total hip replacement (n=30, 57.7%). This was followed by unipolar hemiarthroplasty (n=11, 21.2%), bipolar hemiarthroplasty (n=10, 19.2%) and internal fixation (n=1, 1.9%). Among these patients, six of them had documented complications. There were periprosthetic joint infection (n=2, 3.8%), dislocation (n=2, 3.8%), hematoma formation (n=1, 1.9%) and seroma (n=1, 1.9%). Six months after the surgery, most of the patients were able to ambulate, albeit some patients required walking aid such as walking stick and walking frame. Univariate analysis showed that all the factors were not associated with the complications and the post-operative ambulatory status of the patients.

Conclusion

The incidence of geriatric hip fractures remains high during the COVID-19 pandemic despite the movement control order (MCO) being enforced in Malaysia. With prompt surgical intervention, most of the patients can regain ambulatory status, albeit with a walking aid.

## Introduction

As the population ages, the incidence of osteoporosis also increases accordingly [1-4]. One of the clinical manifestations of osteoporosis is hip fracture [1-5]. With the enhancement of surgical knowledge and advancements in implant materials, effective and proven surgical interventions such as total hip arthroplasty, hemiarthroplasty and implant fixation are available to manage patients with geriatric hip fractures [6-12]. However, due to the fact that all the patients involved are elderly with multiple co-morbidities, patients with geriatric hip fractures are prone to numerous complications such as thromboembolism, infection, gastrointestinal bleeding, delirium and functional loss [10-17]. Approximately 25% of older patients who sustain hip fractures die within one year, and about 13% die within six months after the trauma [5-17]. The recovery rate among those who survived is also poor, with only up to 50% of the patients regaining their ability to perform activities of daily living as prior to the fracture [15-22]. There are many factors that affect the outcomes of geriatric patients with hip fractures. Non-modifiable demographic factors such as age, comorbidities and fracture types can predict the outcomes of these patients [15-22]. Nevertheless, clinicians can optimize the survival and recovery of these patients with prompt surgical intervention, as early surgery for treating geriatric hip fractures reduces mortality and morbidity [15-22].

After the outbreak of coronavirus disease 2019 (COVID-19), not many countries were capable of handling this novel virus (severe acute respiratory syndrome coronavirus 2 (SARS-CoV-2)) [23-25]. On March 12, 2020, the World Health Organization (WHO) declared COVID-19 a pandemic when the number of infected cases exceeded 110,000 in 114 countries [23-25]. Shortly after the declaration of the COVID-19 pandemic, various countries subsequently introduced lockdown measures at different times to stymie the spread of infections [23-25]. In Malaysia, the first lockdown in the form of movement control order (MCO) was introduced on March 18, 2020, in an attempt to reduce the number of infected cases due to limited knowledge of this novel virus and the absence of a definitive cure at that time [23-25]. Nevertheless, the implementation of the MCO and the redirection of healthcare resources to focus on combating COVID-19 came at a cost of collateral damage to other healthcare services [23-25]. Postponement of elective surgeries and delays in treatment were among the difficulties experienced by the public as well as healthcare personnel. Often, these hidden costs of collateral damage remain concealed until much later [23-25]. With this context in mind, our aim is to investigate the surgical outcomes of geriatric patients with hip fractures who were admitted during the initial phase of the COVID-19 pandemic.

## Materials and methods

This was a retrospective study on hip fractures among individuals above 60 years of age. The data of all patients referred to the Joint Replacement Unit at Hospital Kuala Lumpur from March 1, 2020, to March 1, 2021, were retrieved and screened for suitability for this study. All patients who had undergone surgical interventions for hip fractures were included in this study. Ethical approval was obtained from the local medical research and ethics committee.

Demographic data, such as age, ethnicity, gender, side of involvement, mechanism of injury and time interval between initial injury and presentation, were extracted from patients’ medical records by trained site investigators using a customized data collection form. Information regarding treatment, such as the type of surgical intervention and time interval between presentation and surgical intervention, was also gathered from the medical records. The outcomes of the patients were assessed based on their ambulatory status and the incidence of complications, such as infection and dislocation.

The demographic data were presented in a descriptive table. The association between the demographic data and outcomes such as ambulatory status and complications was determined with univariate analysis. The data were analyzed with SPSS version 21 (IBM Corp., Armonk, NY). The patients’ ambulatory status was dichotomized into ambulatory (walking with or without aid, including a wheelchair) and non-ambulatory (bed-bound) for ease of univariate analysis. Similarly, complications were grouped based on their presence or absence. The factors were analyzed with independent t-test, Fisher exact test and Chi-square test for continuous data, factors with two categories and factors with multiple categories, respectively. A p-value of less than 0.05 was considered significant.

## Results

A total of 53 patients were included in this study. The median age of the patients was 72 years with an interquartile range of 12 years (Table 1). There were 32 females (60%) and 21 males (40%) with a female-to-male ratio of 1.5:1 (Table 1). The majority of the patients were Chinese (n=21, 40.4%), followed by Malay (n=14, 26.9%), Indian (n=14, 26.9%) and others (n=3, 5.8%). Thirty patients (57.7%) sustained left-sided neck of femur fractures, while the remaining 22 patients (42.3%) sustained right-sided neck of femur fractures. Three-quarters of the patients sustained the injury due to minimal trauma, with 16 of them sustaining the injury due to slips and miss-steps (each n=16, 30.8%) and eight experiencing dizziness (n=8, 15.4%). Only 12 patients (23.1%) sustained hip fractures due to trauma, such as a fall while exercising (two patients), a fall while doing gardening (one patient), a fall from stairs or escalator (two patients), a fall from road divider (one patient), a fall after a chair broke (one patient) and involvement in motor vehicle accidents (five patients).

**Table 1:** Demographic data of the patients included in this study.

Factors		Number (n)	Frequency (%)	Median	Interquartile range
Age		-	-	72	12
Ethnicity	Malay	14	26.9	-	-
	Chinese	21	40.4	-	-
	Indian	14	26.9	-	-
	Others	3	5.8	-	-
Gender	Male	20	38.5	-	-
	Female	32	61.5	-	-
Side	Right	22	42.3	-	-
	Left	30	57.7	-	-
Mechanism	Dizziness	8	15.4	-	-
	Miss-step	16	30.8	-	-
	Slipped	16	30.8	-	-
	Trauma	12	23.1	-	-
Presentation (day)		-	-	1	1
Time to surgery (day)		-	-	5	4
Surgery	Internal fixation	1	1.9	-	-
	Unipolar hemiarthroplasty	11	21.2	-	-
	Bipolar hemiarthroplasty	10	19.2	-	-
	Total hip replacement	30	57.7	-	-
Complications	Hematoma	1	1.9	-	-
	Seroma	1	1.9	-	-
	Infection	2	3.8	-	-
	Dislocation	2	3.8	-	-
Ambulation status	Walking without aid	16	30.8	-	-
	Walking stick	11	21.2	-	-
	Walking frame	21	40.4	-	-
	Wheelchair	4	7.7	-	-

Most of the patients presented to the hospital early after sustaining the injury, with a median of 1 day and an interquartile range of 1 day. The median time for the interval between presentation and surgery was 5 days, with an interquartile range of 4 days. The majority of the patients were treated with total hip replacement (n=30, 57.7%). This was followed by unipolar hemiarthroplasty (n=11, 21.2%), bipolar hemiarthroplasty (n=10, 19.2%) and internal fixation (n=1, 1.9%). The surgery was decided upon by the arthroplasty surgeon after a discussion with the patient's family members.

A total of six patients (11.3%) experienced complications after their surgeries. These included dislocation (n=2, 3.8%), periprosthetic joint infection (n=2, 3.8%), hematoma (n=1, 1.9%) and seroma (n=1, 1.9%). Six months after their surgeries, most of the patients regained their ambulatory status, albeit some still required walking aids. Sixteen of these patients (30.8%) were able to walk without any assistance. Twenty-one patients (40.4%) were able to walk with a walking frame, and 11 patients (21.2%) were able to walk with a walking stick. Only four patients (7.7%) remained wheelchair-bound despite the surgical intervention. In univariate analysis, no factor was found to be associated with the occurrence of complications (Table 2). Similarly, no factor was noted to be associated with the ambulatory status in this cohort during the univariate analysis (Table 3).

**Table 2:** Association between demographic factors and occurrence of complications. *Independent t-test. **Fisher exact test. #Chi-square test.

Factors		Complication		p value
		No	Yes	
Age (year)		74.07 (7.51)	75.67 (7.63)	0.626*
Ethnicity	Malay	13	1	0.200^#^
	Chinese	17	4	
	Indian	14	0	
	Others	2	1	
Gender	Male	18	2	1.000**
	Female	28	4	
Side	Right	20	2	1.000**
	Left	26	4	
Mechanism	Dizziness	7	1	0.710^#^
	Miss-step	13	3	
	Slipped	15	1	
	Trauma	11	1	
Presentation (day)		22.35 (107.9)	1.17 (0.41)	0.636*
Time to surgery (day)		5.7 (3.04)	6.17 (5.037)	0.743*
Surgery	Internal fixation	1	0	0.873^#^
	Unipolar hemiarthroplasty	9	2	
	Bipolar hemiarthroplasty	9	1	
	Total hip replacement	27	3	

**Table 3:** Association between demographic factors and post-operative ambulatory status of the patients. *Independent t-test. **Fisher exact test. #Chi-square test.

Factors		Ambulatory status		p value
		No	Yes	
Age (mean and standard deviation in years)		76.0 (9.4)	74.1 (7.4)	0.630*
Ethnicity	Malay	2	12	0.713^#^
	Chinese	1	20	
	Indian	1	13	
	Others	0	3	
Gender	Male	1	19	0.565**
	Female	3	29	
Side	Right	1	21	0.629**
	Left	3	27	
Mechanism	Dizziness	0	8	0.274^#^
	Miss-step	2	14	
	Slipped	0	16	
	Trauma	2	10	
Presentation (day)		2.75 (2.9)	21.33 (105.7)	0.729*
Time to surgery (day)		6.0 (1.8)	5.7 (3.4)	0.875*
Surgery	Internal fixation	0	1	0.384^#^
	Unipolar hemiarthroplasty	1	10	
	Bipolar hemiarthroplasty	2	8	
	Total hip replacement	1	29	

## Discussion

Fragility fractures are fractures that occur as a result of mechanical forces that would not typically cause a fracture [1-5]. The most common fragility fractures are hip fractures in elderly patients. These geriatric patients with hip fractures are associated with a high rate of morbidity and mortality [1-2]. Up to 17.7% of these patients pass away due to complications directly or indirectly attributed to hip fractures [1]. Previous studies revealed that early surgical interventions not only significantly reduce the mortality rate in these patients but also improve their quality of life by enhancing functional status. Bretherton and Parker demonstrated that early surgery for patients with hip fractures significantly reduced the 30-day mortality in 6,638 patients over 24 years [21]. This finding has led to the recommendation of surgical intervention within 48 hours for geriatric patients with hip fractures [20]. Similarly, enhanced recovery after surgery (ERAS) programs have been implemented to minimize mortality and morbidity in these patients [20]. ERAS programs emphasize preoperative, intraoperative, and postoperative intervention during the recovery period for geriatric patients [20]. The core principles of ERAS programs are being practiced in our setting, where all geriatric patients with hip fractures are routinely co-managed by a multidisciplinary team, involving medical specialists, rehabilitation physicians, surgeons, anesthetists and supporting staff such as physiotherapists, dieticians, occupational therapists and nurses. The implementation of a hip fracture program has been shown to significantly increase the likelihood of earlier surgery as high-risk, high-need patients can be identified and optimized sooner [20]. In this cohort, the median time to surgery was 5 days with an interquartile range of 4 days. The time to surgery is significantly reduced compared to patients with similar problems in the year 2017 to the year 2020 where the mean time to surgery was 19.8 days [6]. During the study period, amid the COVID-19 pandemic, the number of patients in this cohort (a total of 52 patients) did not differ significantly compared to the years 2017 to 2020 (a total of 168 patients across four years; average of 42 cases per year).

Compared to patients treated in our institution from the year 2017 to the year 2020, patients in this cohort showed improved ambulatory status [6]. The rate of patients who were able to ambulate without walking aids increased from 28.6% to 30.8%. Similarly, upward trends were observed in patients who were ambulating with a walking stick (12.1% to 21.2%) and patients who were ambulating with a walking frame (17.9% to 40.4%). The rate of patients dependent on wheelchairs for ambulation decreased from 26.1% to 7.7%. We postulate that several reasons may contribute to this phenomenon. First and foremost, the treatment approach for geriatric hip fractures shifted toward total hip replacement compared to hemiarthroplasty or internal fixation during the study period. A study of 16,213 patients by Suarez et al. showed that patients who underwent total hip replacement had fewer major and minor complications compared to hemiarthroplasty for the treatment of femoral neck fractures, after controlling for comorbidities [19]. In their study, major complication includes sepsis, septic shock, acute renal failure, pulmonary embolism, ventilation for more than 48 hours, unplanned intubation, myocardial infarction, cardiac arrest, stroke with neurologic deficit and 30-day mortality [19]. The complications that are classified as minor complications are urinary tract infection, pneumonia, superficial surgical site infection, wound dehiscence, progressive renal impairment, deep wound infection, deep vein thrombosis and 30-day readmission [19]. However, univariate analysis within this cohort does not reveal any association between the type of surgery and the occurrence of complications, possibly due to the small sample size (Table 2). Secondly, during the COVID-19 pandemic, these patients were optimized and treated surgically as early as possible to reduce the length of stay in order to preserve the hospital resources to combat COVID-19. Earlier surgical intervention, in turn, facilitated the rehabilitation of these patients. These results are consistent with a report published by Al-Ani et al., which demonstrated that patients who had early surgeries were able to return to independent living at a faster rate than those who underwent delayed surgeries [22].

Limitation

There are several limitations to this observational study. Firstly, this study involved a small sample size with patients from a single center, making it difficult to generalize findings. Secondly, the cross-sectional nature of this study prevents the inclusion of important parameters such as intra-operative details and scoring system for assessment. Nonetheless, this report is the first in our country that investigates the outcomes of geriatric patients with hip fractures treated surgically during the COVID-19 pandemic.

## Conclusions

The incidence of geriatric hip fractures remains high during the COVID-19 pandemic, despite the enforcement of the movement control order (MCO) in Malaysia. During the study period, there is a noticeable trend in the treatment of geriatric patients with hip fractures shifting toward total hip replacement. With prompt surgical intervention, the majority of patients can regain their ambulatory status, although some may require walking aids. Future prospective multicenter studies with larger sample sizes should be conducted to confirm these findings.
